# Bone-muscle crosstalk under physiological and pathological conditions

**DOI:** 10.1007/s00018-024-05331-y

**Published:** 2024-07-27

**Authors:** Yuechao Dong, Hongyan Yuan, Guixing Ma, Huiling Cao

**Affiliations:** 1 Department of Biochemistry, School of Medicine, Guangdong Provincial Key Laboratory of Cell Microenvironment and Disease Research, Shenzhen Key Laboratory of Cell Microenvironment, Key University Laboratory of Metabolism and Health of Guangdong, Southern University of Science and Technology, Southern University of Science and Technology, Shenzhen, 518055 China; 2 Shenzhen Key Laboratory of Soft Mechanics & Smart Manufacturing, Department of Mechanics and Aerospace Engineering, Southern University of Science and Technology, Shenzhen, 518055 China

**Keywords:** Osteogenesis, Myokines, Muscle, Chondrogenesis, Obesity

## Abstract

Anatomically connected bones and muscles determine movement of the body. Forces exerted on muscles are then turned to bones to promote osteogenesis. The crosstalk between muscle and bone has been identified as mechanotransduction previously. In addition to the mechanical features, bones and muscles are also secretory organs which interact closely with one another through producing myokines and osteokines. Moreover, besides the mechanical features, other factors, such as nutrition metabolism, physiological rhythm, age, etc., also affect bone-muscle crosstalk. What’s more, osteogenesis and myogenesis within motor system occur almost in parallel. Pathologically, defective muscles are always detected in bone associated diseases and induce the osteopenia, inflammation and abnormal bone metabolism, etc., through biomechanical or biochemical coupling. Hence, we summarize the study findings of bone-muscle crosstalk and propose potential strategies to improve the skeletal or muscular symptoms of certain diseases. Altogether, functional improvement of bones or muscles is beneficial to each other within motor system.

## Key points

• Bone-muscle crosstalk is established by various osteokines and myokines.

• Bone-muscle crosstalk is subjected to diseases not only defined in motor system.

• MSCs secret exosomes rich in noncoding RNAs to function on muscles.

• Bone metastasis of breast cancer is also harmful to muscles.

## Introduction

The human motor system consists of bones, skeletal muscles, and joints, and is one of nine systems in the human body. Skeletal muscles are capable of autonomous contraction and can move specific parts of the body precisely, making them ideal for directing movement and maintaining posture [1]. In addition to supporting movement, bones and muscles also have other important functions. Bones, skeletal muscles and joints work in conjunction to protect and accommodate visceral organs [2, 3], while calcium and phosphate found in bone matrix maintain demic homeostasis [4]. Muscle tissue is an abundant storage site for glucose and amino acids necessary for proper systemic metabolism [5]. Mesenchymal stem cells (MSCs) and hematopoietic stem cells (HSCs) are produced in bone marrow and differentiate into functional cells [6]. Co-culture of myocytes and chondrocytes promotes the production of cartilage matrix and prevents cartilage injury [7]. Therefore, muscle originated factors have been shown to regulate chondrocyte behaviors.

Research has been dedicated to understanding the mechanism of bone-muscle crosstalk, which plays a cooperative role in body movements. Muscles generate mechanical load that shapes bones and joints, and over 600 proteins secreted by muscles sustain communication with other organs, including bones [8, 9]. Besides, muscular excision can delay fracture healing [10]. Astronauts suffer from skeletal and muscular deficiency due to weightlessness, with muscle recovery taking priority upon returning to Earth, indicating the significance of functional muscles for osteogenesis [11]. While muscles can supply blood to prevent fractures, fascia tissues have a weaker effect on bone than muscles, and thus muscles do not entirely alleviate fractures through blood provision [12]. Muscular reduction also affects metabolic homeostasis, leading to step reduction, which down-regulates the synthetic rate of muscular protein to facilitate insulin resistance and diabetes.

Muscles secrete factors that can affect bones and muscles via paracrine and endocrine signaling, and the same is true for factors from bones [13]. The underlying mechanisms and significance of various factors involved in the interplay between bone and muscle will be introduced in the following context.

## Bone-muscle crosstalk throughout the lifespan

It is well recognized that during embryonic development, there is a tightly coordinated interaction between bones and muscles through the orchestrated effects of the genic network. Muscular contractions play a critical role in determining skeletal status, and this mechanical relationship between bones and muscles persists throughout an individual’s lifetime [14]. Wolff’s law, which proposes that bones can adapt in response to mechanical alteration, has led to the development of Frost’s mechanostat theory, which suggests that bones can maintain their response to exogenous loading within a threshold [15]. Therefore, the load exerted by muscular contractions is a crucial determinant of bone health. In the absence of mechanical load, bones may lose their circular outlines and exhibit worse functional performance. This can lead to deformed long bones because of attenuated bone mineralization and aggravated bone resorption [16]. In healthy adults, a lack of movement or weightlessness can result in the loss of bone mass. Muscular mass generally peaks at around 30 years of age, after which it begins to decline. By the time they reach the age of 70, their muscle mass may only be retained at 60-80% of their peak levels [17]. Additionally, bone mineral density (BMD) is known to decline starting at around age 50. In addition to the natural metabolic decline of bones and muscles, physical inactivity and unloading can exacerbate the loss of BMD and muscle mass in the elderly, regardless of the presence of any disease [18]. Consequently, research on the bone-muscle crosstalk can contribute to enhancing the physical fitness of older adults and potentially delay the aging process.

## Factors derived from muscles act on bones via paracrine signaling

### Fibroblast growth factor

Fibroblast Growth Factor (FGF) is crucial to different stages of OB differentiation. In MSCs, the differentiation orientation is determined by the type of FGFR. The FGF/FGFR1 signal in MSCs directs the differentiation of OBs lineage [19]. Within skeletal system, FGF have been shown to promote osteoblast (OBs) differentiation through the activation of the Phosphoinositide 3-kinase (PI3K)/AKT pathway and the production of Runt-related transcription factor 2 (Runx2) and Osterix [20]. Inactive phosphorylated Glycogen Synthase Kinase 3β is decreased in reduced differentiating OBs derived from *FGF2*^−/−^ mice with lower BMD, resulting in reduced nucleus β-catenin, which can be partially reversed by exogenous FGF2 [21]. Thus, mesoporous silica nanoparticles have been designed as a sustained release vector of FGF2, which has shown promising results in bone regeneration [22]. Additionally, the chondrocyte differentiation of MSCs is directed to generate endochondral ossification, the process involves proliferation, differentiation, hypertrophy, and apoptosis of chondrocytes [23]. Activation of FGFR2 in MSCs leads to differentiation into proliferative chondrocytes expressing FGFR3, which subsequently develop into hypertrophic chondrocytes expressing FGFR1. As a result, FGF pathways play a crucial role in all stages of entochondrostosis [24]. Aside from the deficiency in osteogenesis, MSCs derived from FGF2 null mice are also more prone to adipogenesis. The FGF2/ Extracellular signal-regulated kinase (ERK) signal triggers the nuclear localization of transcriptional co-activator with PDZ-binding motif and its interaction with Runx2 in MSCs, which leads to their differentiation into osteoblasts instead of adipocytes. Hence, FGF2 has the potential to inhibit adipogenesis as well [25]. Additionally, it has been discovered that the side effects of FGF2 on fibro/adipogenic progenitors in aging muscles actually favor the miR-29a/SPARC axis. As a myokine and adipogenic inhibitor, SPARC is targeted by an increase in miR-29a, leading to the generation of intramuscular adipose tissues (ATs) [26]. Therefore, the impact of FGF2 on the weakened bone-muscle crosstalk in aging individuals remains uncertain. Altogether, FGF signals promotes osteogenesis, chondrogenesis and inhibits adipogenesis to positively regulate skeletal system. And in musculoskeletal system, sarcolemma is injured by mechanical actions to release FGF and the loading stimulation can be transformed into myogenic response via autocrine [27]. In muscle-bone interfaces, FGF can also interact with receptors on periosteum to promote the muscle-bone interaction [28]. Interval intramuscular injection of FGF2 in rats further reinforces the bone morphogenetic protein 2 (BMP2)-induced osteogenesis [29]. Therefore, FGF2 also amplifies other osteogenic signals such as BMP2 and Wnt/β-catenin, resulting in synergistic effect. FGF2 was confirmed to function on the BME of humans. Physiologically, FGF2 alleviates the injury caused by strengthened physical work via promoting osteogenesis, cartilage regeneration and even repairs fracture [30] (Table 1).

**Table 1:** Diseases regulated by factors from bones and muscles

Factors	Molecular type	Target tissues/cells	Associated Diseases	
FGF2	Growth factors	OBs Chondrocytes	Administration of FGF2 ameliorates impaired bone structure in rat models of osteoporosis through PI3K/Akt/Runx2 signaling pathway [149]. Additionally, FGF2 treatment mitigates bone loss in mice by downregulating SCL expression [185]. Elevated FGF2 is detected in the chondrocytes of individuals with osteoarthritis, leading to the aggravation of cartilage degradation through the induction of MMP13 and ADAMTS5 production [168].	
FGF21	Growth factors	BMMCs BMMs Osteoclasts	Increased FGF21 in mice with muscular dystrophy results in defective osteogenesis and strengthened adipogenesis [151]. FGF21 promotes bone resorption via RANKL-induced osteoclastogenesis [151]. Elevated FGF21 is detected in mice with osteoporosis, leading to the enhancement of RANKL/RANK/NFATc1 signaling pathways [150].	
IGF	Growth factors	OBs	Combination between IGFs and IGFR is inhibited in individuals with lower BMD [44]. RR ameliorates BME of diabetic rats via enhancing IGF1/PI3K/mTOR signaling pathways [152]. Lactoferrin improvements of the aging BME of mice is IGF1-dependent. Conversely, Nicotine induces bone dysplasia in embryos and newborns of rats through the downregulation of IGF1 [40, 153].	
Serum IL6	Myokines	OBs MSCs/Chondrocytes	Postmenopausal osteopenia treated by bisphosphonate and calcitriol results in decreased serum IL6 [65]. In RA patients, Tocilizumab inhibits soluble IL6R to decrease the production of osteoclastic factors produced by OBs to alleviate the syndromes [175]. Chondrogenesis of MSCs is initialized through IL6/STAT3/SOX9 axis. In chondrocytes, IL6 promote chondrogenesis via STAT3/BMP4. IL6 deficiency mice suffer from the joint inflammation and bone loss caused by OA [66, 174] .	
IL7	Myokines	T cell Synoviocyte/ Chondrocyte	IL7 upregulates RANKL expression by T cells and promotes osteoclastic differentiation of B cells in the bone marrow. Elevated IL7 in the serum and bone of OVX models is responsible for bone resorption [70, 71]. In synoviocyte of RA patients and chondrocytes of OA patients, IL7 probably impairs cartilage tissues via promoting the production of more MMPs [172, 173].	
IL15	Myokines	NK cells	In RA patients, more M-CSF and RANKL are expressed by NK cells after being stimulated with IL15. The differentiation of monocytes into OCs is enhanced. In contrast, IL15R deprivation in mice increases the bone mass [171].	
Irisin	Myokines	OBs Adipocytes Chondrocytes	Circulating Irisin in patients with diabetic osteopenia is low [78]. Irisin decreases the fracture risk in postmenopausal women and prevents bone loss of astronauts in microgravity environment [158, 159]. Irisin induces browning of WAT in mice with prediabetic syndromes [78]. Irisin alleviates OA symptoms in both mice and human patients through inhibiting NF-κB and (NLRP3)/caspase1pathways [86, 165, 170].	
Myostatin	Myokines	Bones/muscles Myocytes Satellite cell	Decreased Myostatin restores insufficient osteogenesis and myogenesis in mice^105^. To decrease Myostatin in patients, injection of ActRIIB was performed on postmenopausal women to decrease Myostatin, which results in an increase in lean mass and osteogenic biomarkers [105]. Maternal deficiency of Myostatin is detrimental to the bones of newborn mice. Myostatin is probably responsible to inherited osteopenia [101]. In RA mice, Myostatin inhibition mitigates arthritis symptoms via inhibition of bone destruction [93]. Myostatin suppresses IGF pathway in cachexia. Restriction of Myostatin in mice increase the survival rate of cachexia [186].	
Osteoactivin		Myocytes	Osteoactivin-enriched mice exhibit enhanced production of MMPs which remodel fibrotic ECM. Ultimately, the process of muscular fibrosis induced by distracting osteogenesis shows attenuation [111].	

*ActRIIB* Class II TGFβ transmembrane receptor, *ADAMTS5* A disintegrin and metalloproteinase with thrombospondin motifs 5, *BMD* Bone mineral density, *BME* Bone microenvironment, *BMMCs* Bone marrow-derived mesenchymal stem cells, *BMMs* Bone marrow macrophages, *BMP4* Bone morphogenetic protein 4, *ECM* Extracellular matrix, *FGF* Fibroblast growth factor, *FST* Follistatin, *HO1* Heme oxygenase 1, *IGF* Insulin-like growth factor, *IL* Interleukin, *IL6R* Interleukin 6 receptor, *IL15R* Interleukin 15 receptor, *MMP13* matrix metalloproteases, *MSCs* Mesenchymal stem cells, *M-CSF* Macrophage colony-stimulating factor, *NFATc1* Nuclear factor of activated T cells, *Nrf2* Nuclear factor erythroid 2-related factor 2, *NK cells* Nature Killer cells, *OA* Osteoarthritis, *OBs* Osteoblasts, *OCs* Osteoclasts, *OVX* Ovariectomy, *PI3K* Phosphoinositide 3-kinase, *RA* Rheumatoid arthritis, *RANK* Receptor activator of nuclear factor-kappa B, *RANKL* Receptor activator of nuclear factor-kappa B ligand, *rIGF1* recombinant Insulin-like growth factor, *Runx2* Runt-related transcription factor 2, *RR* Rehmanniae Radix Praeparata, *SCL* Sclerostin, *SOX9* sex-determining region Y-box transcription factor 9, *STAT* Signal transducer and activator of transcription,

In this case, FGF2 involved in bone-muscle crosstalk targets on SCL, the Wnt antagonist, to prevent bone loss and sustain skeletal homeostasis via Myostatin inhibition [30]. Therefore, FGF2 has potential therapeutic applications in bone-related diseases such as severe osteoporosis that are not responsive to present therapy and bone defects. However, the use of exogenous FGF2 can lead to uncontrollable release and adverse effects. It remains further exploration to design rational administration.

FGF21 is majorly expressed in liver and adipose tissues, which are closely related to skeletal system. Insulin-like growth factor binding protein (IGFBP1), which is induced by FGF specifically in the liver, can bind to integrin β1 through its RGD domain in hematopoietic progenitors to promote osteoclasts (OCs) differentiation and bone resorption stimulated by Receptor activator of nuclear factor-kappa B ligand/Receptor activator of nuclear factor-kappa B (RANKL/RANK) signaling [31]. FGF21 potentiates the activity of peroxisome proliferator activated receptor γ (PPARγ) to direct the differentiation of bone marrow mesenchymal stem cells (BMMSCs) into adipocytes instead of osteoblasts. In contrast, mice with a functional mutation of FGF21 exhibit a high bone mass phenotype due to scarce PPARγ [32]. Additionally, FGF21 directly suppresses the proliferation, differentiation, and growth hormone sensitivity of isolated chondrocytes from the growth plates of mice by binding to FGFR1/3 [33]. Overall, FGF21 has been shown to have deleterious effects on bone metabolism, but the exact mechanism requires further investigation. Intriguingly, muscles also produce FGF21, which aids in glucose uptake through autocrine signaling [34]. However, the roles of FGF21 in bone muscle crosstalk remains examination.

### Insulin-like growth factor 1

Insulin-like growth factor 1 (IGF1) is primarily localized on skeletal muscle adjacent to the periosteum, which allows it to be recognized by the IGF1R that is richly expressed in periosteum. The production of IGF1 is higher in skeletal muscles that undergo exercise. Additionally, IGF1 plays a role in other pathways that underlie osteogenesis [35]. For example, the promotion effect of BMP9/Smad on the osteoblastic differentiation of MSCs is further enhanced by exogenous IGF1 [36]. IGF also promotes the RANKL production in OBs to regulate OCs [37]. Overall, the osteogenic ability of IGF1 is superior to bone resorption [38]. In response to IGF1, the bone resorption sites mold the architecture for osteogenesis to form the integrated cycle of bone remodeling [39].

In contrast, low level IGF is detrimental to skeletal development and cartilage structure at different ages. Injecting pregnant rats with nicotine subcutaneously can lead to the downregulation of IGF and matrix in embryonic growth plates. This can cause a delay in skeletal development in embryos and weight loss in newborns, partially explaining how maternal smoke exposure can harm fetuses [40]. Neonatal Mice lacking IGFR1 in cartilage, which results in defective chondrogenesis, have lower body weight, poor skeletal mineralization, and a short lifespan after birth [41]. Definitely, chondrogenic alteration is also influenced by the interaction of IGF/IGFR with other pathways. When rats are fed a soft diet after weaning, the levels of IGFs and IGF receptors in their masseter muscles are decreased and results in the morphologic alteration of mandible [35]. In MSCs, chondrocyte differentiation is positively affected by the combined effects of IGF1 and Transforming growth factor (TGFβ1), which provides a theoretical foundation for stem cell therapy in cartilage lesions [42]. Indian hedgehog (Ihh) also promotes parathyroid hormone related protein to retain proliferative chondrocytes in the growth plate, leading to the continuous production of more Ihh. However, this positive feedback loop is prevented by IGF/IGF1R, and mature chondrocytes expressing Ca^2+^-sensing receptors (CaSR) emerge. Mature chondrocytes express CaSR first to mediate direct actions of Ca^2+^ on terminal differentiation via IGF/IGF1R [43]. Clinically, on account of inhibiting the binding between IGFs/IGFR to reignforce bone resorption, IGF binding protein 2 (IGFBP2) is the most dependable predictor in the serum of individuals with lower bone mineral density, regardless of their age and gender [44]. In conclusion, the bone-protective effects of IGF have been widely studied in vivo and provide a prospective theoretical basis for future clinical trials.

### Interleukins

**Fig. 1:**
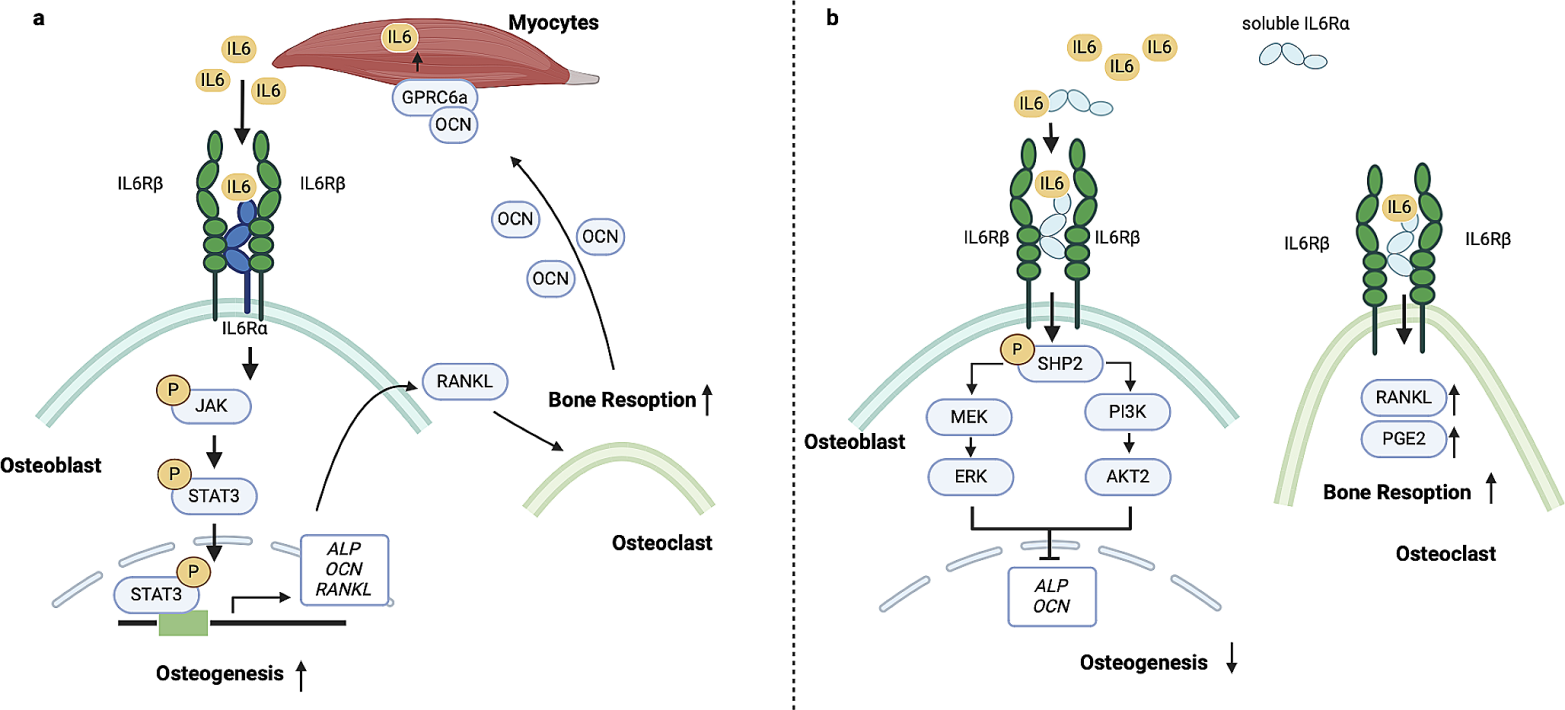
The osteogenic effect depends on the origination and receptor type of IL6. (**a**) After the recruitment of gp130 (IL6Rβ), membrane anchored receptors (IL6Rs) combine with muscular IL6 to positively regulate osteogenesis through JAK/STAT3 signaling pathway in osteoblasts. RANKL production in OBs is also increased by muscular IL6 to mediate bone resorption. Then, more OCN released by the dynamic bone tissues functions on GPRC6A in muscles to produce more IL6. (**b**) Circulating IL6 binds the soluble receptors (sIL6Rs) to negatively regulate osteogenesis through SHP2/PI3K/AKT2 or SHP2/MEK/ERK signaling pathways in osteoblasts. Meanwhile, osteoclastic RANKL and PGE2 is increased to accelerate bone resorption. *ERK* Extracellular signal-regulated kinase, *gp130* glycoprotein 130, *GPRC6A* G protein-coupled receptor family C group 6 member A, *IL* Interleukin, *IL6R* Interleukin 6 receptor, *JAK* Janus kinase, *MEK* mitogen-activated protein kinase-extracellular signal–regulated kinase kinase, *OBs* osteoblasts, *OCN* osteoclasts, *PGE2* Prostaglandin E2, *PI3K* Phosphoinositide 3-kinase, *RANK* Receptor activator of nuclear factor-kappa B, *RANKL* Receptor activator of nuclear factor-kappa B ligand, *SHP2* Src-homology domain 2 containing protein-tyrosine phosphatase, *sIL6R* soluble Interleukin 6 receptor, *STAT3* Signal transducer and activator of transcription 3

Relaxation and contraction of muscles alter the level of Interleukin 6,7 and 15 to affect bone homeostasis. Overall, IL6 is primarily synthesized by the liver to promote inflammation and insulin resistance [45]. Particularly, mechantisms of secreted IL-6 are determined by origination. Circulating IL-6 comes from fibroblasts, keratinocytes, mesangial cells, vascular endothelial cells, mast cells, macrophages, dendritic cells, T cells, and B cells [46]. IL-6 produced by macrophages and fibroblasts functions through juxtacrine mechanisms [47, 48]. IL-6 produced by dendritic cells [49], Fibroblasts [50], Keratinocytes [50], mesangial cells [51], B cells [52], T cells [53] and endothelial cells [54] through paracrine mechanisms. Additionally, IL-6 produced by mast cells functions through Autocrine mechanisms [55]. However, in muscular tissues, IL6 is produced acutely in response to mechanical activity and is beneficial for anti-inflammation and glucose uptake, making it a myokine [56]. Excessive exercise results in increased production of IL6, which induces muscle hypertrophy after regeneration of impaired positions [57]. Moreover, IL-6 is involved in the bone muscle crosstalk. Muscle-derived IL6 binds to IL6 receptors on OBs, leading to the phosphorylation of Janus kinase (JAK) and signal transducer and activator of transcription (STAT), which subsequently triggers an increase in osteoblastic markers, such as osteocalcin (OCN) and alkaline phosphatase [58]. OCN, originating from bone tissue, interacts with G protein-coupled receptor class C group 6 member A (GPRC6A) receptors located in myofibers, resulting in enhanced expression of the myokine IL6. This upregulation of IL6 further stimulates bone resorption by promoting the production of RANKL from OBs. Consequently, the heightened bone resorption process leads to an increased release of OCN from the bone matrix. This virtuous circle facilitates adaption to exercise by inducing nutrient catabolism [59]. On the other hand, mice lacking muscular IL6 exhibit poor exercise capacity, which can be improved by injecting OCN into their muscles. Interestingly, the deficiency of IL6R in OBs, rather than myofibers, reduces the muscular response to exercise, similar to IL6 deficiency [60]. However, in contrast to IL6 derived from muscles, circulating IL6 decreases bone mass by functioning on both OBs and OCs. As depicted in Fig. 1, the contradictory roles of IL6 in osteogenesis can be attributed to two pathways: Classic IL6 binds to IL6Rα located on cellular membrane, recruiting a homodimer of glycoprotein 130 (IL6Rβ). Trans*-*signal IL6 binds to soluble IL6Rα to activate cells lacking IL6R [61]. Therefore, the coexistence of IL6 and sIL6R in circulating serum is responsible for the loss of bone mass. Soluble IL6R is necessary to amplify IL6 effects due to the poor IL6R on the membrane of OBs [62]. However, activation of sIL6R inhibits osteogenesis via the ERK or PI3K/AKT2 pathway mediated by the phosphorylation of Src-homology domain 2 containing protein-tyrosine phosphatase (SHP2) [63]. And in OCs, production of RANK and prostaglandin E2 (PGE2) is also driven by activation of sIL6R to facilitate bone resorption [64]. Due to their dual reinforcement of bone resorption, the effects of circulation IL6 are chronic and detrimental to bone-muscle system. Therefore, bone turnover in mice with high levels of IL6 is hampered due to both impaired osteogenesis and bone resorption. Although their serum IL6 levels decreased as their BMD and handgrip strength improved, IL6 levels did not change further after 6 months of therapy [65], indicating that there is no direct correlation between serum IL6 levels and treatment response. IL6 also plays a role in triggering chondrogenesis, with phosphorylation of Signal transducer and activator of transcription 3 (STAT3) leading to the differentiation of MSCs into chondrocytes by increasing and activating BMP4 [66]. Thus, the contradictory effects of IL6 on bone homeostasis are determined by the underlying pathophysiological conditions.

While human myocytes do express and secrete IL7, satellite cells with high differentiation potency exhibit increased levels of IL7 mRNA and protein. Consequently, terminated myotubes exhibit significantly lower expression of IL7R mRNA. IL7 signal has the potential to sustain the phenotype of SCs, which is evident in its ability to facilitate cellular migration and the expression of *PAX7*, a marker of SCs that prevents myogenesis [67]. As a mediator of the acquired immune system, IL7 upregulates RANKL, which is derived from T cells and promotes osteoclastic differentiation [68, 69]. Inhibition of IL7 has a pro-osteogenesis effect indicated by increased serum level of OCN [70, 71].

In mice, an increase in circulating IL15 levels following exercise or genetic modification was found to enhance bone mass and mineralization [72]. This increase in IL15 also stimulated OBs to produce more RANKL, which mediates bone resorption, and tumor necrosis factor α, which mediates inflammation. In rodents, IL15 has been shown to play a role in the early stages of OC differentiation [73]. IL15-treated Nature killer (NK) cells also promoted OBs apoptosis, ultimately leading to increased bone resorption [74]. Conversely, in mice deprived of IL15R, bone mass was reinforced due to the inhibition of immune cell-dependent osteoclastogenesis [75]. Overall, IL15 plays a complex role in regulating bone resorption and bone mass.

Moreover, inappropriate exercise causes inflammation events of muscles and bones, which may accompany with altered Interleukins. As the potential therapeutic targets, ILs involved in bone muscle crosstalk remains further research to improve inflammatory reactions.

### Irisin

Irisin is derived from fibronectin type III domain containing 5 (FNDC5), which is a downstream factor of PPARγ coactivator 1α (PGC1α) and is secreted by muscle tissue after being modified and spliced [76]. Overall, Irisin enhances insulin sensitivity in various systems, including livers, muscles and adipose tissues [77]. Irisin was administered to mice with prediabetic symptoms, leading to activation of genes that facilitate the conversion of white adipose tissue (WAT) to brown adipose tissue, thereby optimizing the body’s metabolic state [78]. The thermogenic effect of brown adipose tissue was further enhanced by high doses of Irisin, while low doses of Irisin were found to increase bone mass in the skeletal system. Accumulated research indicates that Irisin promotes the differentiation and activity of OBs lineage. During the osteoblastic differentiation of MSCs, Irisin binds to the membrane anchored receptor αV integrin, resulting in the phosphorylation of ERK/STAT3 cascades that induce BMP2/Smad signaling [79]. Irisin also promotes osteogenic differentiation of MSCs by activating autophagy via the Wnt/β-catenin signal pathway [80]. In addition, Irisin exerts multiple inhibitory actions on the TGFβ pathway, thereby favoring osteogenesis in OBs. Mitogen-activated protein kinases (MAPKs) signals alleviate TGFβ1-induced inhibition of Runx2, and Irisin also competitively binds to TGF-β type II receptor [81]. Finally, Irisin upregulates osteogenic genes, such as osteopontin (OPN) to induce osteoblastic differentiation. Exogenous Irisin administration was found to improve bone mass and induce osteogenesis in animal models. The exact mechanism of action in vivo remains unclear, although the involvement of MAPK has been suggested [82]. Irisin also stimulates the release of SCL and RANKL by osteocytes via α_V_β_5_ integrin receptor. Irisin also directly stimulates OCs function [83], but for the osteoclast precursor cells, it only promotes cell proliferation while inhibiting differentiation [84].

In addition to OBs, the pro-osteogenic effect of Irisin has been observed in various cell types. Irisin treatment has been shown to induce phosphorylation of AMP-activated protein kinase α in macrophages, leading to a shift from M0/M1 phase to M2 phase, and promoting osteogenesis by secreting anti-inflammatory factors [85]. In view of this, in vitro studies also showed that irisin treatment of chondrocytes from osteoarthritis (OA) patients resulted in a superior anabolism-to-catabolism ratio [86].

Clinically, exercise have been recognized as the first line for the treatment and prognosis of diseases such as diabetes, obesity and inflammations, etc. Serum Irisin was found to be elevated by the contracted muscles to function on bone metabolism via factor such as Sclerostin, Leptin and Brain-derived neurotrophic factor. The beneficial effect of exercise on pathological bones is at least partly derived from Irisin [87].

### Myostatin

Myostatin, also known as growth and differentiation factor 8, is the most potent inhibitor of muscle development. Studies have shown that mice lacking myostatin exhibit hypertrophic muscles and higher bone mineral density [9]^,^ [88]. Therefore, the significance of Myostatin was also established in bone homeostasis. In bones, the activation of transcription factors such as Nuclear factor of activated T cells (NFAT) and (cAMP)-response element-binding protein by TGFβ or IGF1 determines the expression of myostatin within bones [89]. Myostatin has negative effects on OBs by activating ERK1/2 and TGFβ, and repressing the Wnt pathway [90, 91]. Osteocytes secrete miR-218 in exosomes to promote differentiation of bone marrow cells into OBs by activating the Wnt signal. Myostatin suppresses miR-218 and accelerates the production of Sost, RANKL, and Dickkopf (DKK1). Thus, Myostatin also acts as a positive regulator of osteoclasts [92]. The role of myostatin in osteoclasts was first described by Berno Dankbar et al [93]. P-Smad2, a downstream target of myostatin, can promote osteoclastic differentiation through interacting with NFATc1 [94]. However, it is possible that Myostatin’s ability to inhibit bone formation may be more pronounced in BMMSCs rather than in OBs or OCs. BMMSCs treated with myostatin are prone to adipogenic differentiation. Increased myostatin level in obese individuals can be reversed by the caloric limitation. Inhibition of myostatin can lead to reduced AT and strengthened bones and muscles [95], but the direct roles of myostatin on bones is still unknown.

As expected, osteogenic functions can be improved by Myostatin inhibition. Class II TGFβ transmembrane receptor (ActRIIB) is a soluble decoy receptor of myostatin located on the membrane of OBs. ActRIIB-Fc fusion protein which binds myostatin could increase osteogenesis indicated by elevated serum Type I procollagen and BMP without affecting muscles in mice, suggesting a direct effect of ActRIIB on OBs [96, 97]. ActRIIB is also expressed by chondrocytes, and myostatin-deficient chondrocytes have been shown to increase cartilage area by promoting SOX5/9 in the fracture callus, suggesting that myostatin may play a role in endochondral ossification [98]. Furthermore, Myostatin deficiency was extensively studied in mice with a range of skeletal disorders. In RA mouse models lacking myostatin, there is less bone destruction, indicating that myostatin could be a potential target for RA treatment [93]. Endurance exercise has been shown to promote the production of follistatin (FST) and its related factors, which inhibit myostatin. FST has been found to alleviate myostatin-induced bone degeneration in Type 2 diabetes mellitus (T2DM) mice [99]. Fstl3 null mice are more prone to fractures and loss of osteocyte mechanosensitivity [100]. Maternal deficiency of myostatin alters the calvarial collagen of newborn mice, resulting in permanent lesions in the skeletal architecture of adult offspring. Targeting myostatin pathways in the gestational environment provides a potential therapeutic approach for inherited osteopenia [101]. Pulsed ultrasound therapy for fracture recovery is probably based on the osteogenic effect of myostatin inhibition [102].

Clinically, Myostatin is only detectable in fractured tibias, not normal ones, and may reduce the secretion of citrate - a necessary component for osteogenesis - by inhibiting the osteogenic differentiation of MSCs through IGF1 dependent signaling pathway. NADPH oxidase 4, downstream effector of Myostatin-Smad2/3 signaling pathway, generates ROS that can arrest cell cycle progression and lead to apoptosis in MSCs [103]. Inhibiting Myostatin activity in MSCs can improve their osteogenic ability, however, this improvement can be negated by microgravity [104]. Both the lean mass and osteogenic biomarkers were significantly elevated in postmenopausal women injected with ActRIIB for one month [105]. Although the poteintial functions of Myostatin in bone muscle crosstalk has been proposed, the muscular and skeletal reinforcement of deficient myostatin failed to prevent the catabolism during unloading, indicating that myostatin’s participation in bone-muscle crosstalk requires further investigation.

### Factors control transdifferentiation of myoblasts into osteoblasts

Transmembrane protein 119 (Tmem119) is a transmembrane protein that plays a crucial role in the differentiation of myoblasts. Rather than differentiating into myotubes, Tmem119 promotes differentiation of C2C12 cells into osteoblasts by inducing the expression of osteogenic markers such as Runx2 and Osterix. BMP2 promotes differentiation of myoblasts into OBs through binding to BMP type 1 receptor expressed by myoblasts. Tmem19 is stimulated by BMP signal in myoblasts and in turn amplifies BMP signal via enhancing ATF4 expression to generate positive feedback loop [106]. Moreover, Tmem119 has been shown to mediate the known osteogenic effect of PTH/Smad3 significantly. Along with its augmented effect on BMP2/Runx2 in myoblasts, Tmem119 plays a critical role in promoting the differentiation of myoblasts into osteoblasts [107]. Osteoactivin was firstly discovered in an osteopetrotic rat through mRNA differential display and distributed in the OBs, skeletal muscle, etc. Under unloading conditions, the elevation of muscular osteoactivin levels prompts myoblasts to differentiate into OBs [108]. During the process of bone mineralization, the BMP2/Smad1 pathway enhances ALP activity in OBs through osteoactivin [109]. Otherwise, the effect of osteoactivin on muscular fibrosis has also been verified. Osteoactivin also triggers matrix metalloproteases 3/9 (MMP3/9) production in fibroblasts, but not in myocytes, when muscles undergo unloading treatments like denervation or tail-suspension [110]. When the tibiae of osteoactivin-transgenic (OA-Tg) mice were distracted, fibrotic ECM was remodeled by increasing MMPs to reduce muscular fibrosis caused by distraction-induced osteogenesis [111]. During muscular fibrosis, fibroblasts repair injured muscles and generate scar tissues with exudative fibrositis. Consequently, weaker myodynamia and elasticity threaten muscular health and Osteoactivin may be the therapeutic target.

## Factors derived from bone tissues that act on muscles in a paracrine manner

### Prostaglandin E2

When exposed to fluid shear stress, osteocytes can release prostaglandin E2 (PGE2) through Connexin 43 (Cx43) hemichannels. PGE2 activates the β-catenin pathway directly by increasing PI3K in OBs [112]. Additionally, IL6/STAT3 can stimulate the production of PGE2 in OBs, which promotes the differentiation and activation of OCs [113]. Ultimately, PGE2 plays a crucial role in bone turn over and the proliferation and differentiation of myoblasts with unknown mechanisms, making it potentially important for muscle myogenesis [114].

### Sclerostin

Sclerostin (SCL), encoded by *SOST gene*, is a negative regulator of bone formation and its neutralizing antibody is effective in reducing the incidence of osteoporosis [115, 116]. Osteocytes exhibit mechanosensitive behavior, enabling the bones to respond appropriately to varying degrees of stimulation. Changes in SCL expression are observed during loading and unloading of the bones [117]. When subjected to unloading stimuli, mature osteocytes produce SCL, which acts on the Wnt/β-catenin pathway to inhibit osteogenesis. Definitely, bone derived SLC also influences musculoskeletal system. SCL binds to low-density lipoprotein receptor‐related proteins 5 and 6, which are co-receptors of Wnt. Wnt3a induces upregulation of MyoD and Myogenin expression in myoblasts in muscle tissue, which promotes differentiation, but this process can be impeded by SCL [118]. Treatment with SCL enhances phosphorylation of mechanistic target of rapamycin (mTOR) in C2C12 cells, leading to endoplasmic reticulum stress by inhibiting autophagy. On the other hand, TGFβ1 causes muscle fiber atrophy through Nuclear factor κB (NF-κB) and p38 signaling, and increases Pax7-positive satellite cells, which can be reversed by SCL neutralizing antibody [119]. Altogether, functional myoblasts can be disturbed by SCL, which is the potential target of improving abnormal muscles.

### FGF23

Parathyroid hormone (PTH) initiates the release of calcium from bones into the bloodstream and promotes the reabsorption of calcium ions in renal tubules. Additionally, PTH inhibits the reabsorption of phosphate. FGF23, the first hormone identified in skeletal tissues, is secreted by osteoblasts and osteocytes to regulate calcium and phosphorus levels by regulating PTH. FGF23 also inhibits the activation of vitamin D and renal 1α hydroxylase. Conversely, PTH stimulates FGF23 production in OBs to promote proliferation instead of differentiation [120]. Removing the parathyroid gland reduces the circulating levels of FGF23. FGF23 exacerbates the depletion of calcium and phosphorous in the skeletal system. It’s worth noting that FGF23 functions on skeletal muscles, smooth muscles and cardiac muscles. Mechanistically, Klohto functions as the soluble co-receptor of FGF23 in plasma and regulates skeletal muscle differentiation by inhibiting the Insulin/IGF1 signal [12]. Additionally, FGF23 has effects on smooth muscle and myocardial tissue. FGF23/Klohto induces vascular contraction by increasing ROS production in smooth muscle cells. However, Klohto also increases endothelial NO, which partially alleviates vessel constriction. Chronic kidney diseases (CKD) results from decreased levels of Klotho and increased levels of FGF23 in the serum [121]. FGF23 also plays a role in cardiovascular events by regulating cardiac muscle. Specifically, bone-derived FGF23 has a paracrine pattern and induces left ventricular hypertrophy by activating the calcineurin/NFAT signal in cardiomyocytes [122]. Due to abnormal intracellular Ca^2+^ levels, FGF23-induced hypertrophy of the myocardium can lead to systolic dysfunction and even arrhythmia [123, 124]. Altogether, FGF23 functions as bridge molecules cross-link motor system to circulation system. Research around FGF23 is potential to further decipher the improvement of cardiovascular disease through exercise.

### Osteocalcin

OCN is a type of non-collagenous protein primarily expressed by OBs. Uncarboxylated osteocalcin (ucOCN), an isoform of OCN, acts as an endocrine hormone and binds to GPRC6A receptors on myocytes. This interaction promotes myocyte proliferation through either the PI3K/Akt or MAPK pathways and myocyte differentiation via the Erk1/2 signal [125]. Insulin-dependent glucose uptake is also enhanced by ucOCN following muscular contraction by increasing GPRC6A levels and virtuous circle contibutes to functional muscles has generated [126].

However, mice with special mutation of Cx43 in OBs/osteocytes shows decreased circulating OCN and weaker muscles, but normal glucose levels. Synthetic OCN is able to partially restore muscle strength in these mice [127]. Mice with specific deficiency of GPRC6A exhibit defective glucose intake, fat metabolism and IL6 production, etc. Conversely, the absence of tyrosine phosphatase, which acts as an inhibitor of GPRC6A, led to the opposite phenotype [128].

OCN has been found to be effective in improving muscle function and reversing muscular aging under various adverse conditions. When young mice were given OCN, they exhibited stronger athletic performance. Aged mice treated with OCN, the decline in endurance could be reversed [129]. Mice injected with OCN intraperitoneally could produce more IL6 by their muscles, which phosphorylates STAT3 and induces autophagy of myocytes, potentially inhibiting muscular aging. OCN treatment could also increase muscle mass and strength in aging mice [130]. ucOCN also increases insulin sensitivity and improves glucose intake in mice [131]. Experiments with mice treated with slow-release corticosterone showed that exogenous ucOCN can help recover circulating hormones and glucose uptake in the muscles, alleviating the serious side effects of corticosterone on the musculoskeletal system [132]. Overall, OCN treatment shows great potential as a strategy for treating muscular diseases of unknown etiology.

### IGF1

IGF1, produced by OBs or osteocytes, activates PI3K/Akt pathway in skeletal muscles [133]. IGF1 derived from osteocytes is indispensable for loading-induced Wnt/β-catenin in the OBs lineage [134]. Conditional knockout of IGF1 in mice also reduced IGF1 in bones and muscles together [135]. Thus, IGF serves as a bridge for bone-muscle communication (Table 2).

**Table 2:** Diseases regulated by factors from bones

Factors	Molecular Type	Target tissues/cells	Associated diseases
FGF23	Osteoblast-derived molecules	Myocytes	Antagonizing FGF23 enhances muscular strength to ameliorate hypophosphatemia and rickets in juvenile XLH mice [179].
Sclerostin	Osteocyte-secreted molecules	Myocytes	Orchiectomy-induced mice exhibit elevated serum SCL levels, contributing to the development of compromised bone and muscle strength [118].
Osteocalcin	Osteoblast- derived molecules	Myocytes	Aging muscles are strengthened by intraperitoneal injection of OCN in mice through activating IL6/STAT3 signaling pathway [130]. Osteogenesis imperfecta patients express much less circulating ucOCN and OCN [155]. Reduced OCN in postmenopausal women is responsible for muscular loss [156].

*FGF* Fibroblast growth factor, *IL* Interleukin, *OCN* Osteocalcin, *SCL* Sclerostin, *STAT3* Signal transducer and activator of transcription, *ucOCN* Uncarboxylated osteocalcin, *XLH* X-linked hypophosphatemi

## Exosomal non-coding RNAs serve as the molecular intermediaries for the crosstalk between muscle and bone

Exosomes are small vesicles, typically ranging in diameter from 40 to 100 nm, that have the ability to bind to cellular membranes. Upon fusion with membranes, exosomes are released by cells into the extracellular space and can subsequently be endocytosed by other cells. Due to their cargo of proteins and nucleic acids, exosomes play critical roles in mediating both cell-to-cell and organ-to-organ interactions [136]. In 2010, Guescini et al. reported the production of exosomes by muscle cells are effective within muscular system, which is manifested as myoblast and myocytes utilizing exosomes in the range of 50–80 nm to communicate with each other [137]. Moreover, loading muscles secrete more exosomes into circulation, where they can act on distant targets, such as skeletal tissues [138].

The exosomes derived from myoblasts are known to possess high level of miR-27a-3p, which functions to selectively target adenomatous polyposis coli, thereby promoting the activation of β-catenin and ultimately facilitating the maturation of OBs [139]. Conversely, exosomes sourced from aging myoblasts harbor miR-34a, which acts to repress the differentiation of MSCs [140]. Within osteocytes, it has been observed that the exosomes originating from myocytes can initiate the activation of the Wnt/β-catenin pathway, which is accompanied by the concurrent suppression of osteocyte apoptosis [92].

In recent years, there also has been a growing body of evidence indicating that exosomes originating from skeletal tissues exert significant effects on muscle physiology. Compared with other cells within skeletal tissues, these exosomes are majorly derived from MSCs. Similarly, the role of miR-215 in muscular metabolism has been investigated, with exosomes from MSCs modified to overexpress this microRNA being found to protect against myoblast apoptosis through the miR-215/fatty acid binding protein 3 pathway [141]. Furthermore, intramuscular injection of MSC exosomes has been found to ameliorate inflammation and fibrosis in skeletal muscle contusions, thereby promoting muscular healing [142]. This effect is at least partially mediated by the polarization of macrophages towards an anti-inflammatory M2 phenotype by the exosomes [143]. Finally, it has been demonstrated that MSC exosomes containing higher levels of SirT1 can promote the activation, proliferation, and differentiation of satellite cells, suggesting their potential therapeutic utility in muscular regenerative medicine [144]. MSCs-derived exosomes are potential to alleviate side effects of clinical medicines on muscles. Specifically, the miR-486-5p found within MSC exosomes has been shown to act on Forkhead Box O1 (FOXO1), inhibiting both its nuclear localization and the expression of genes associated with muscular atrophy. This inhibition can effectively counteract the effects of dexamethasone-induced muscular relaxation. To further illustrate the potential therapeutic applications of exosomes in muscular pathology, mice were treated with busulfan-cyclophosphamide to induce cachexia-like conditions. These mice exhibited increased levels of circulating activin A and decreased muscular mass, reminiscent of cancer cachexia patients. treatment with exosomes derived from tonsil-derived MSCs, which are enriched in miR-145-5p, was found to effectively rescue muscular atrophy by targeting the activin A receptors ACVR2A and ACVR1B on myoblasts [145].

Although exosomes derived from MSCs have been extensively studied, the muscular effects of OB-derived exosomes were only proposed last year. Exosomes from femoral OBs contain higher levels of taurine upregulated 1 (TUG1) and lower levels of differentiation antagonizing nonprotein coding RNA (DANCR), which are long noncoding RNAs. TUG1 promotes the nuclear translocation of myogenic factor 5 to the promoter of the *myogenin* gene, while DANCR exerts the opposite effect. By contrast, exosomes from skull OBs contain decreased TUG1 and increased DANCR [146]. The regulatory effects of exosomal long noncoding RNAs from OBs on muscle mass are region dependent. Exosomes present and function in skeletal tissues are also subject to regulation by muscles indirectly. Within MSCs, miR-218 targets Wnt signal inhibitors DKK2, SCL, and Secreted Frizzled Related Protein 2, thereby enhancing Wnt-induced osteogenesis. Conversely, osteocytes treated with muscle-derived Myostatin exhibit decreased miR-218 expression and increased levels of DKK1, SCL, and RANKL, which are packaged into exosomes and internalized by osteoblasts (OBs). As a result, Wnt signaling is inactivated, leading to the abolition of OB differentiation [92].

**Fig. 2:**
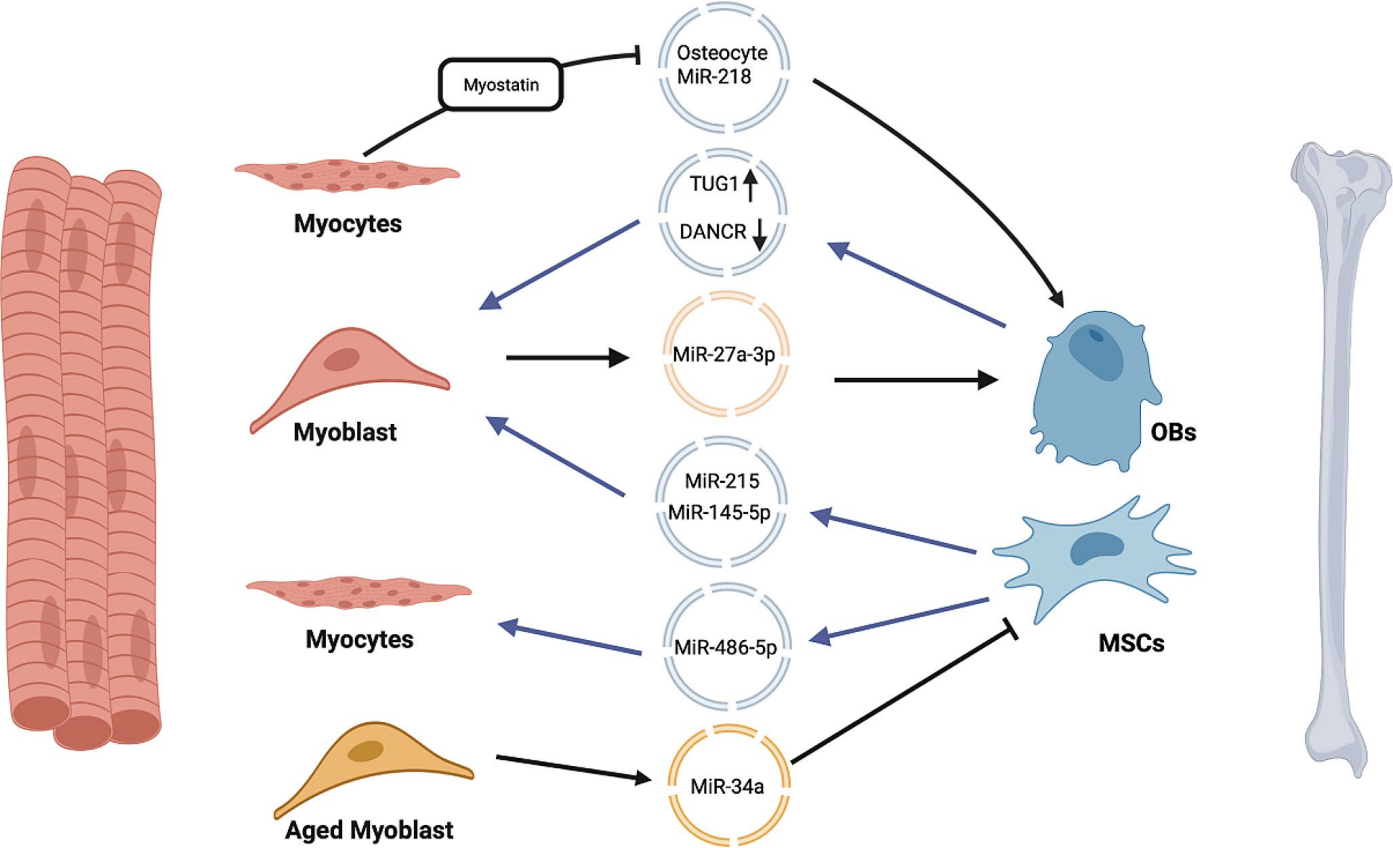
The role of exosomes in bone-muscle crosstalk. Exosomes are responsible for transporting noncoding RNAs between bones and muscles. Myoblasts promote osteoblastic differentiation through releasing exosomes rich in miR-27a-3p, and aging myoblasts prevent MSCs through releasing exosomes rich in miR-34a. Osteocytes release exosomes rich in miR-218 to promote OBs differentiation. Myostatin derived from muscles decreases miR-218 in osteocytes to prevent OBs differentiation. In turn, MSCs secrete exosomes rich in miR-486-5p and miR-215/miR-145-5p to positively regulate myocytes and myoblasts differentiation respectively. OBs produce exosomes rich in LncRNA TUG1 and lack in DANCR to mediate the differentiation of myoblasts. *DANCR* Differentiation antagonizing nonprotein coding RNA, *LncRNA* Long no coding RNA, *MSCs* Mesenchymal stem cells, *OBs* Osteoblasts, *TUG1* Taurine upregulated 1

In summary, noncoding RNAs packaged in exosomes establish interactions between bones and muscles (Fig. 2). and moreover, exosomes-mediated paracrine or juxtacrine mechanisms within BME have also been studied [147]. Being rich in miR-214, OCs secrete exosomes which colocalize with OBs intraosseously and lead to attenuated activity. Osteoclastic exosomes contain miR-214 bridge the linkage between OCs and OBs through ephrinA2-EphA2 interaction. Finally, enhanced bone resorption and anti-osteogenic process have been embodied in OVX mice and old women with osteoporotic fracture [148]. Being extensively presented in organisms, exosomes are stable, easy to store and available to diagnose diseases in diverse stages. Compared with tissue biopsy, it can reduce the patient’s sampling pain.

## Bone-muscle crosstalk in various diseases within skeletal system

**Fig. 3:**
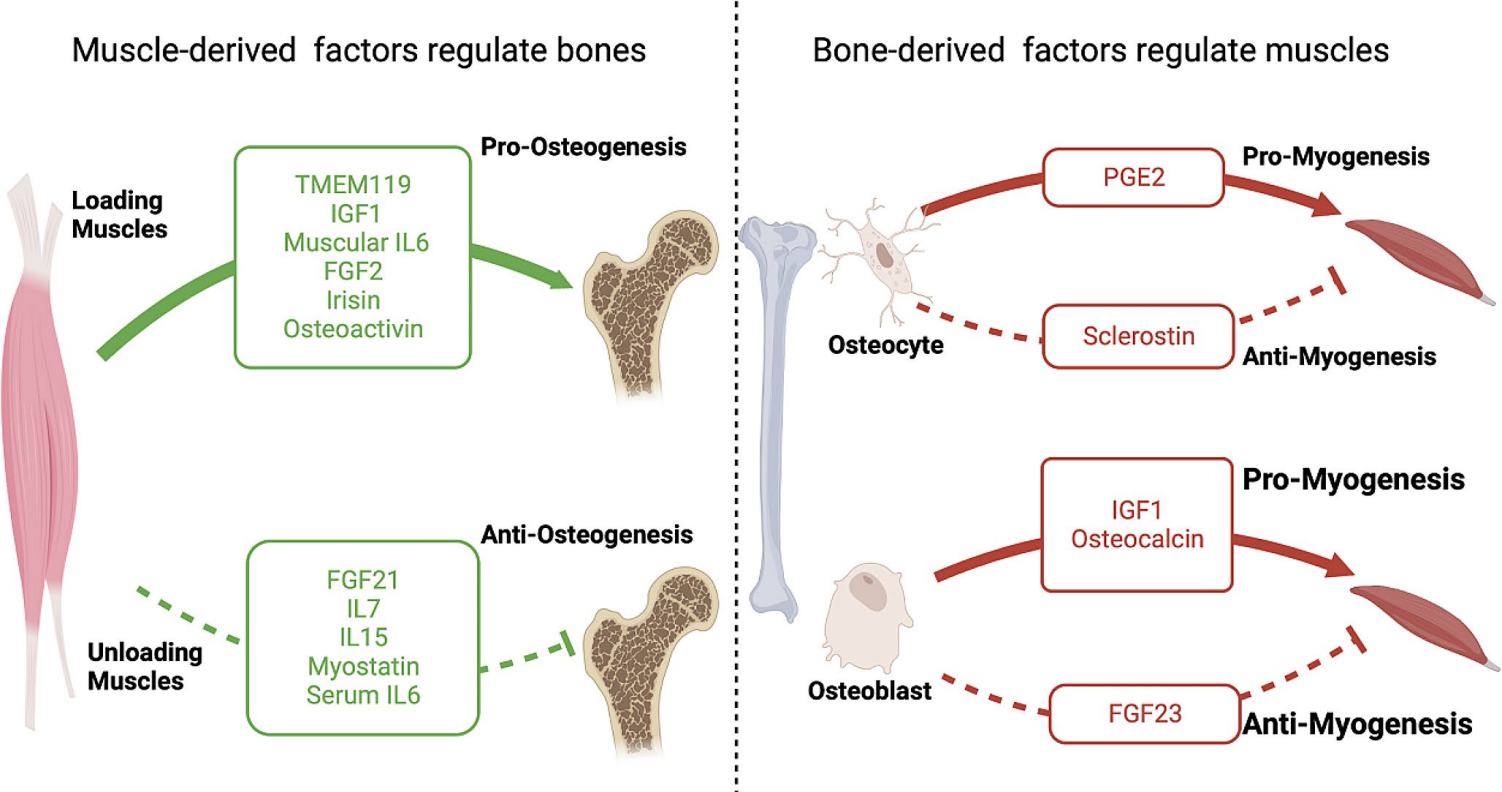
The role of muscle derived factors and bone derived factors in bone-muscle crosstalk. Muscle derived factors such as IGF, FGF, ILs, Myostatin, Irisin, etc., regulate bone homeostasis. In turn, bone derived factors such as PGE2, FGF23, Osteocalcin, Sclerostin, Osteoactivin, etc., regulate myogenesis. *FGF* Fibroblast growth factor, *IGF* Insulin-like growth factor, *IL* Interleukin, *PGE2* Prostaglandin E2, *TMEM119* Transmembrane protein 119

Bones and muscles interact each other through secreting various factors (Fig. 3). Therefore, complicated network between bones and muscles has also been examined in diseases of skeletal system.

### Osteoporosis

Osteoporosis (OP) is primarily driven by an imbalance between bone resorption and osteogenesis. In view of the conclusions from animal experiments, Factors originating from muscular tissues are also believed to have the potential to influence the progression of osteoporosis. Intraosseous administration of exogenous FGF2, a muscle-derived osteogenic growth factor, has demonstrated its efficacy in enhancing bone formation in ovariectomized (OVX) rats with osteoporosis [149]. Moreover, in the serum of OVX, muscle-derived FGF21 and IL-7 are elevated to aggravate bone resorption and decrease bone volume, which can be alleviated by miR-100-5p and IL-7 neutralization respectively [70, 150]. Similarily, increased serum FGF21 and decreased FGF21 in the liver and is also observed in mice with muscular dystrophy. However, FGF21 secretion is decreased in the liver and WAT leading to impaired osteogenesis, increased bone resorption, and adipogenesis [151].

Existing medicines also improve osteoporosis of OVX models through targeting muscle-derived factors and the downstream cascades. Rehmanniae radix praeparata (RR), a traditional Chinese medicine, encompasses bioactive compounds that promote the proliferation and differentiation of pre-osteoblast cells via activation of the IGF1/PI3K/mTOR signaling pathway. Additionally, RR extract has demonstrated efficacy in ameliorating the bone microenvironment (BME) in diabetic rats [152]. Consequently, RR holds significant potential as a viable therapeutic candidate for diabetic osteoporosis. Lactoferrin (LF) has been reported to improve the BME in aging mice and prevent OB senescence, as evidenced in in vitro experiments. This beneficial effect of LF is attenuated when interfering with IGF1 using small interfering RNA (siRNA), implying that LF’s actions in treating aging-related osteoporosis may be mediated through the IGF1 pathway [153]. Importantly, it is noteworthy that IGFBP5 exhibits the capability to reverse osteogenic deficiencies in mice subjected to ovariectomy (OVX), suggesting a plausible IGF1-independent mechanism of action [154].

Clinical studies have provided evidence indicating that individuals diagnosed with osteogenesis imperfecta and experiencing progressive deformity exhibit reduced levels of circulating ucOCN and OCN. This decline in ucOCN and OCN levels is also associated with compromised muscular functions in comparison to patients presenting with milder to moderately severe phenotypes of the condition [155]. Furthermore, postmenopausal women display a decline in muscle mass, which correlates with reduced OCN levels [156]. Additionally, postmenopausal women with impaired osteogenesis show elevated levels of serum IL6, a pro-inflammatory cytokine, and a combination therapy involving Bisphosphonate and Calcitriol has shown promise in enhancing bone mineral density (BMD) [65]. Notably, patients with diabetic osteopenia exhibit low levels of circulating Irisin [157]. Additionally, Irisin has been demonstrated to offer fracture risk prevention in postmenopausal women [158] and help counteract bone loss in astronauts exposed to a microgravity environment [159].

In summary, OP can be worsened by impaired muscular function, leading to a progressive decline in muscle health. This creates a detrimental cycle that illustrates the interplay between bone and muscle events during the development of osteoporosis. Undoubtedly, the secretion of factors by muscles exhibits a strong correlation with OP, a relationship well-supported by robust clinical research. Nevertheless, the in-depth pre-clinical investigation into the effects of IGF1 and FGF21 on OP offers promising avenues for subsequent clinical research endeavors.

### Osteoarthritis

Osteoarthritis (OA), predominantly affects the elderly population, is characterized by the progressive degradation of cartilage and a decrease in its synthesis, which is considered the principal underlying mechanism. Besides their well-known osteogenic capabilities, muscle-secreted factors also exert an influence on chondrogenesis, holding promise for the regulation of events related to OA. In OA mouse models, the muscle-secreted factor FGF21 can serve as a target to ameliorate symptoms through preventing diverse programmed death of chondrocytes. Nuclear translocation of the transcription factor EB further facilitated increased FGF21-induced autophagy flux and activated the SIRT1-mTOR cascade, alleviating extracellular matrix (ECM) proteolysis, chondrocyte apoptosis, and senescence, and thereby improving OA in murine models [160]. Additionally, interferon regulatory factor 7 (IRF7), known as a negative regulator of FGF21, was found to be upregulated in LPS-stimulated chondrocytes, mediating pyroptosis. And the absence of IRF7 alleviated OA in mice by upregulating FGF21 expression [161]. Moreover, the inflammatory response of chondrocytes in the OA model can also be regulated by muscular-derived factors. It has been observed that Harpagoside can effectively suppress the expression of IL6 in primary chondrocytes from human OA [162]. Moreover, blockage of IL6 and retinoic acid receptor-related orphan receptor-α, upstream regulator of IL6/Stat3, alleviates OA mice [163, 164]. In murine models, intra-articular injection of Irisin led to a significant reduction in OA symptoms, which could be attributed to improved mitochondrial membrane potential, suppressed mitophagy, and reduced inflammation-induced reactive oxygen species (ROS) production [165]. In rabbit models of OA, IGF1 demonstrated protective effects on hyaline cartilage by inhibiting NF-κB signaling and ROS production. Consequently, IGF1 treatment both in vitro and in vivo restrained inflammatory and apoptotic responses in chondrocytes [166]. Interestingly, the adult offspring of prenatal nicotine exposure showed increased susceptibility to OA, with the underlying mechanism involving decreased IGF1 levels due to the activation of α4β2-nicotinic acetylcholine receptors in articular cartilage [167].

Contrarily, in OA patients, chondrocytes exhibit increased levels of FGF2, which stimulates the upregulation of MMP13 and a disintegrin and metalloproteinase with thrombospondin motifs 5 (ADAMTS5) via specific binding to fibroblast growth factor receptor 1 (FGFR1). This molecular cascade ultimately leads to the degradation of cartilage by reducing proteoglycan levels [168]. Furthermore, increased circulating IL6 induced by unloading muscle triggers deleterious reactions within the body. Notably, the level of IL6 in the synovial fluid of OA patients correlates with the inflammatory progression of the disease [169]. In OA patients, moderate exercise was found to ameliorate cartilage damage by stimulating the secretion of muscular irisin, which, in turn, inhibited the NF-κB pathway and NOD-like receptor family pyrin domain containing 3 (NLRP3)/caspase1 activation, thereby preventing pyroptosis in chondrocytes [170].

Collectively, multiple muscular factors have the potential to influence the progression of OA, and the strengthening of muscles can play a role in mitigating OA by improving the condition of cartilage tissues.

### Rheumatoid arthritis

In individuals diagnosed with Rheumatoid Arthritis (RA), the condition is characterized by the presence of an inflamed synovium, which leads to the development of painful, stiff, and swollen symmetrical joints, predominantly in the hands and feet. The synovium in RA patients is characterized by inflammatory responses and exhibits an abundance of interleukins, which are secreted by functionally impaired muscles. In the synovial fluid of RA patients, the presence of IL15 stimulates natural killer (NK) cells to express higher levels of M-CSF and RANKL, thus promoting monocyte differentiation into osteoclasts (OCs) [171]. This process contributes to bone degradation within the affected joint cartilage, a common observation in conditions like OA or RA. Another important player in the pathogenesis of OA and RA is autocrine IL7, which, when present in joint cartilage tissues, promotes the destruction of articular cartilage by upregulating the levels of MMPs [172, 173]. In pathological conditions similar to those observed in OA, IL6-deficient mice exhibited reduced proteoglycan levels and cartilage erosion [66, 174]. In summary, interleukins derived from muscles mediate the inflammation in RA, thereby destroying bone and cartilage tissues of lesions.

Current therapeutic approaches primarily focus on ameliorating inflammatory responses and always causes significant side effects. Identifying potential therapeutic targets becomes crucial, and certain muscle-derived factors with inflammatory properties have been found to be abundant in synovial tissues, exacerbating symptoms associated with RA. For RA patients, treatment with Tocilizumab, an IL6 antibody drug targeting the IL6 receptor (IL6R), has shown promising outcomes, including a decrease in osteoclastic markers and an overall improvement in clinical symptoms [175]. Therefore, maintaining muscular functions is possible to alleviate the inflammatory responses of RA.

### Intervertebral disc degeneration

The intervertebral disc is a complex structure composed of three main components: the nucleus pulposus, annulus fibrosus, and cartilaginous endplate The protrusion of the intervertebral disc occurs due to degenerative changes in these components [176]. Specifically, in intervertebral disc degeneration (IVDD), the NP may protrude through the damaged annulus fibrosus, leading to compression of adjacent spinal nerves, resulting in symptoms like waist ache and lower limb numbness. To counteract these adverse effects, physical exercise has been shown to elevate Irisin levels both in NP and circulation. Irisin produced by loading muscles activates autophagy in NP tissues, offering a potential means of mitigating IVDD. In an IVDD rat model, Lentivirus expression of FNDC5 specifically in the central area of NP mimics the beneficial effects of physical exercise. Conversely, Irisin knockdown (KD) abolishes the mitigative effect of exercise on IVDD [177]. Mechanistically, Irisin treatment downregulates the phosphorylation of large tumor suppressor and Yes-associated protein 1 while upregulates Connective tissue growth factor in cultured nucleus pulposus cells (NPCs), thus promoting the anabolism in NPCs and inhibiting catabolism of ECM [178]. Overall, while still in its primary stages, future research exploring the biological interactions between muscle and IVDD holds promising potential.

### Other skeletal diseases

Abnormal levels of FGF23, a factor secreted by osteoblasts and osteocytes, have been observed in metabolic disorders such as hypophosphatemia and rickets. In the context of rickets, inadequate mineralization of the bone matrix primarily results from persistent hypophosphatemia. Notably, studies have demonstrated that administering anti-FGF23 treatment can ameliorate hypophosphatemia and rickets in young mice with X-linked hypophosphatemia (XLH), while also increasing muscle strength and spontaneous motor activity in adult XLH mice [179].

### Bone-muscle crosstalk in response to massage

Strenuous exercise leads to delayed onset muscle soreness, which are manifested as muscular swelling and reduced muscle performance within the first 24 h. Based on a meta-analysis, massage alleviates soreness and contributes to muscular recovery 24 h post-exercise [180]. Clinical trials also revealed enhanced osteogenic effect of massage in postmenpusal women [181]. In order to further improve pathological bones such as ostogenesis in premature infant or chronic neck pain, massage should accompany with physical activity [182, 183]. In conclusion, passive force of muscles brought by massage is also beneficial to bone metabolism. Making certain the exact mechanisms of bone-muscle crosstalk during massage is available for establishing the rehabilitation scheme of pathological bones or muscles.

### Exercise-intervention in metabolic diseases

Being characterized by appetite stimulator. Asprosin was initially identified as a hunger hormone. Unstrained women during the menstrual cycle have higher circulating Asprosin than trained women. Asprosin is also elevated in women after anaerobic exercise. In contrast, aerobic exercises serum Asprosin is significantly decreased. Not being affected by glucagon or epinephrine, the novel hormone Asprosin contributed to the release of hepatocyte glucose, the metabolism of which is involved in the hyperglycemia amelioration effect of exercise. Aerobic exercise decreases the hepatic Asprosin level by PKA/TGF-β pathway to alleviate impaired glucose metabolism. By virtue of 3 glycosylation sites, Asprosin promotes myotubular GLUT4 or phosphorylates muscular AMPK to uptake glucose. Collectively, Asprosin may be crucial to metabolic disorders, such as diabetes and obesity, which can be improved by exercise [184].

## Conclusion

Throughout the lifetime, the architecture and functions of bones and muscles are closely intertwined, demonstrating the bi-directional communication between mechanical and biological factors from embryonic development to aging.

Further investigation into the relationship between the musculoskeletal system and other bodily systems is necessary to uncover additional mechanisms and develop new strategies for combating diseases related to the musculoskeletal system. This will expand our understanding of the complex interplay between bones and musclesand may ultimately lead to more effective treatments for certain diseases.

## Data Availability

All data of this study are available within the article or the supplementary materials. All data are available from the corresponding authors upon reasonable request.

## Abbreviations

ActRIIB: Class II TGFβ transmembrane receptor
ADAMTS5: A disintegrin and metalloproteinase with thrombospondin motifs 5
AT: Adipose tissue
BME: Bone microenvironment
BMMSCs: Bone marrow mesenchymal stem cells
BMP: Bone morphogenetic protein
CaSR: Ca^2+^-sensing receptors
Cx43: Connexin 43
DANCR: Differentiation antagonizing nonprotein coding RNA
DKK: Dickkopf
ECM: Extracellular Matrix
ER: Endoplasmic reticulum
ERK: Extracellular signal-regulated kinase
FEV1: Forced expiratory volume
FGF: Fibroblast growth factor
FNDC5: Fibronectin type III domain containing 5
FST: Follistatin
GPRC6A: G protein-coupled receptor family C group 6 member A
HO1: Heme oxygenase 1
IGF: Insulin-like growth factor
IGFBP: Insulin-like growth factor binding protein
Ihh: Indian hedgehog
IL: Interleukin
IRF7: Interferon regulatory factor 7
IVDD: Intervertebral disc degeneration
JAK: Janus kinase
LF: Lactoferrin
MAPKs: Mitogen-activated protein kinases
MBD: Mineral and Bone Disorder
MMP: Matrix metalloproteases
MSCs: Mesenchymal stem cells
mTOR: Mechanistic target of rapamycin
NF-κB: Nuclear factor κB
NFAT: Nuclear factor of activated T cells
NLRP3: NOD-like receptor family pyrin domain containing 3
NP: Nucleus pulposus
NPCs: Nucleus pulposus cells
Nrf2: Nuclear factor erythroid 2-related factor 2
OA: Osteoarthritis
OBs: Osteoblasts
OCN: Osteocalcin
OCs: Osteoclasts
OPN: Osteopontin
OVX: Ovariectomy
PGE2: Prostaglandin E2
PI3K: Phosphoinositide 3-kinase
PPARγ: Peroxisome proliferator-activated receptor γ
PTH: Parathyroid hormone
RA: Rheumatoid arthritis
RANK: Receptor activator of nuclear factor-kappa B
RANKL: Receptor activator of nuclear factor-kappa B ligand
rGH: Recombinant Growth hormone
ROS: Reactive oxygen species
RR: Rehmanniae Radix Praeparata
Runx2: Runt-related transcription factor 2
SCL: Sclerostin
SGLT2: Sodium-glucose cotransporter 2
SHP2: Src-homology domain 2 containing protein-tyrosine phosphatase
SOCS3: Suppressors of cytokine signaling3
STAT: Signal transducer and activator of transcription
T2DM: Type 2 diabetes mellitus
TGF: Transforming growth factor
Tmem119: Transmembrane protein 119
TUG1: Taurine upregulated 1
ucOCN: Uncarboxylated Osteocalcin
UPS: Ubiquitin-proteasome system
WAT: White adipose tissue
XLH: X-linked hypophosphatemia
